# Macrophage Markers Do Not Add to the Prediction of Liver Fibrosis by Transient Elastography in Patients With Metabolic Associated Fatty Liver Disease

**DOI:** 10.3389/fmed.2020.616212

**Published:** 2020-12-18

**Authors:** Konstantin Kazankov, Chiara Rosso, Ramy Younes, Angelo Armandi, Hannes Hagström, Holger Jon Møller, Per Stål, Elisabetta Bugianesi, Henning Grønbæk

**Affiliations:** ^1^Department of Hepatology and Gastroenterology, Aarhus University Hospital, Aarhus, Denmark; ^2^Institute of Liver and Digestive Health, University College London, London, United Kingdom; ^3^Department of Medical Sciences, Division of Gastroenterology and Hepatology, University of Turin, Turin, Italy; ^4^Boehringer Ingelheim International, Gesellschaft mit beschränkter Haftung, Ingelheim, Germany; ^5^Department of Upper GI, Unit of Hepatology, Karolinska University Hospital, Stockholm, Sweden; ^6^Department of Medicine, Clinical Epidemiology Unit, Solna, Karolinska Institutet, Stockholm, Sweden; ^7^Department of Medicine, Huddinge, Karolinska Institutet, Stockholm, Sweden; ^8^Department of Clinical Biochemistry, Aarhus University Hospital, Aarhus, Denmark

**Keywords:** macrophages, cirrhosis, biomarkers, NAFLD, Fibroscan

## Abstract

**Background and Aims:** Non-invasive fibrosis staging is essential in metabolic associated fatty liver disease (MAFLD). Transient elastography (TE) is a well-established method for liver fibrosis assessment. We have previously shown that the macrophage marker sCD163 is an independent predictor for fibrosis in MAFLD. In the present study we tested whether the combination of macrophage markers and TE improves fibrosis prediction.

**Methods:** We measured macrophage markers soluble (s)CD163 and mannose receptor (sMR) in two independent cohorts from Italy (*n* = 141) and Sweden (*n* = 70) with biopsy-proven MAFLD and available TE.

**Results:** In the Italian cohort, TE and sCD163 showed similar moderate associations with liver fibrosis (rho = 0.56, *p* < 0.001 and rho = 0.42, *p* < 0.001, respectively). TE had an area under the Receiver Operating Characteristics curve (AUROC, with 95% CI) for fibrosis; F ≥ 2 = 0.79 (0.72–0.86), F ≥ 3 = 0.81 (0.73–0.89), F4 = 0.95 (0.90–1.0). sCD163 also predicted fibrosis well [F ≥ 2 = 0.71 (0.63–0.80), F ≥ 3 = 0.82 (0.74–0.90), F4 = 0.89 (0.76–1.0)]. However, combining sCD163 and TE did not improve the AUROCs significantly [F ≥ 2 = 0.79 (0.72–0.86), F ≥ 3 = 0.85 (0.78–0.92), F4 = 0.97 (0.93–1.0)]. In the Swedish cohort, TE showed a closer association with fibrosis (rho = 0.73, *p* < 0.001) than sCD163 (rho = 0.43, *p* < 0.001) and sMR (rho = 0.46, *p* < 0.001). TE predicted fibrosis well [F ≥ 2 = 0.88 (0.80–0.97), F ≥ 3 = 0.90 (0.83–0.97), F4 = 0.87 (0.78–0.96)], whereas sCD163 did not (best AUROC 0.75). sMR showed a better prediction [F ≥ 2 = 0.68 (0.56–0.81), F ≥ 3 = 0.82 (0.71–0.92), F4 = 0.79 (0.66–0.93)], but the addition of sMR did not further improve the prediction of fibrosis by TE.

**Conclusion:** In these cohorts of MAFLD patients, TE was superior to macrophage markers for fibrosis prediction and in contrast to our hypothesis the addition of these markers to TE did not improve its predictive capability.

## Introduction

Metabolic associated fatty liver disease (MAFLD), a newly instituted and more appropriate term for non-alcoholic fatty liver disease (NAFLD) (1), is an increasingly prevalent liver condition estimated to affect a quarter of the world's population with even higher prevalence in the constantly growing group of subjects with obesity and type 2 diabetes (2). The majority of patients with MAFLD do not develop significant liver disease, however, some of them may progress to cirrhosis and liver failure ultimately warranting a liver transplant. In this respect, the stage of fibrosis has repeatedly proved to be the most important determinant of outcome in MAFLD patients (3, 4), making accurate identification of patients with advanced fibrosis crucial in the management of MAFLD. So far, liver biopsy remains the gold standard for fibrosis staging in MAFLD, however it is invasive and thus not suitable for general use given the vast numbers of subjects in need of examination. Therefore, a number of non-invasive tools for fibrosis assessment have been established (5).

Transient elastography (TE) provides the value of liver stiffness as a measure of fibrosis. It has shown good accuracy in MAFLD, particularly for advanced fibrosis and cirrhosis detection (6), and has been introduced into the practical guidelines for MAFLD diagnosis and management (7). However, TE has several limitations including imperfect prediction (8). Therefore, combinations of TE with other markers have been explored, for instance, a recent multi-center study developed and validated a score consisting of TE, controlled attenuation parameter (CAP) and aspartate aminotransferase (AST) to identify MAFLD patients with inflammatory activity, steatohepatitis and significant fibrosis (9).

Macrophages play an important role in MAFLD (10), and we and others have shown good predictive capability of the macrophage specific marker soluble (s)CD163 for fibrosis in MAFLD (11, 12). Furthermore, the addition of sCD163 to the established NAFLD fibrosis score (NFS) improved its performance (13). Another macrophage marker, the soluble mannose receptor (sMR), is associated with acute and chronic liver disease (14–16) and has not been investigated in adult MAFLD before.

We hypothesized that combining the macrophage markers sCD163 or sMR with TE would result in improvement of fibrosis prediction, and we tested this hypothesis in two independent cohorts of MAFLD patients with biopsy-proven disease.

## Methods

### Study Population

This cross-sectional study was performed in two established cohorts of MAFLD patients from liver centers in Italy and Sweden. The Italian cohort consisted of patients from the Division of Gastroenterology and Hepatology, Department of Medical Sciences, University of Torino, Italy, while the Swedish cohort comprised patients included at the Karolinska University Hospital, Stockholm, Sweden. Both of these cohorts have been described in previously published reports (13, 17).

At both sites, all patients were referred for the investigation of abnormal liver tests or steatosis detected by ultrasound, and MAFLD was diagnosed by liver biopsy. In the Italian cohort, all patients had an alcohol intake of <20 g/day assessed by interviews with the patient or close family members, and in the Swedish cohort an intake of <30 g/day (males) an <20 g/day (females), assessed with phosphatidylethanol (PEth) and the alcohol-use disorders identification test (AUDIT) and the lifetime drinking history (LDH) questionnaires (18, 19).

Liver disease of other etiology was excluded. The number of patients with biopsy-proven MAFLD and available TE was 141 in the Italian cohort and 70 in the Swedish cohort. At the time of liver biopsy, demographic and clinical data were recorded, including age, gender, ethnicity, height, weight, and waist circumference. Body Mass Index (BMI) was calculated. Diabetes was defined as hemoglobin A1c ≥ 48 mmol/mol, fasting blood glucose ≥7.0 mmol/L, previous diagnosis of diabetes or use of anti-diabetic drugs.

At the time of biopsy, a fasting blood sample was obtained and routine biochemical tests were performed. Additional blood samples were drawn and frozen at −80°C for future research. All patients signed an informed consent form in accordance with the Helsinki Declaration. The acquisition, storage, and use of blood samples were approved by the Ethics Committee of the University Hospital San Giovanni Battista of Torino and by the Regional Ethics Committee of Stockholm (2011/13-31-/1 and 2018/134-32).

### Biochemical Analyses

Liver and hematological parameters, fasting glucose and insulin, triglycerides and cholesterol and its components were determined using standard assays and methods. All sCD163 and sMR measurements were performed at the Department of Clinical Biochemistry, Aarhus University Hospital.

This was done in duplicate by in-house enzyme-linked immunosorbent assays (ELISAs) using a BEP-2000 ELISA-analyzer (Dade Behring) essentially as previously described (20, 21). Soluble CD163 and sMR are both resistant to repeated freezing and thawing (20–22). Control samples and serum standards were included in each run to avoid bias. We have previously established reference intervals for sCD163 (0.69–3.86 mg/L) and sMR (0.10–0.43 mg/L) in large cohorts of healthy individuals using the same assays (21, 23).

### Histological Analysis

Liver biopsies were stained and examined locally by experienced pathologists as described by Kleiner et al. (24) and in accordance with the Fatty Liver Inhibition of Progression (FLIP) algorithm for the diagnosis of steatohepatitis (25). All biopsies had a minimum of 11 portal tracts, and inadequate biopsies were excluded.

### Transient Elastography

Vibration controlled transient elastography (FibroScan, Echosens, Paris, France) was performed within 2 weeks prior to the liver biopsy by expert operators in accordance with the instructions by the manufacturing company, including at least 3 h of fasting. The M probe was used as standard, and the XL probe as per the automatic probe selection tool or when the M probe failed.

TE was expressed in kilopascal (kPa) and calculated as the median value of 10 successful acquisitions, defined by a success rate of >60%, and by an interquartile range <30%.

### Calculation of the NAFLD Fibrosis Score and FIB-4

The NFS was calculated using the existing formula: −1.675 + 0.037 × age (years) + 0.094 × body mass index (kg/m^2^) + 1.13 × impaired glucose tolerance/diabetes mellitus (yes = 1, no = 0) + 0.99 × aspartate aminotransferase/alanine aminotransferase – 0.013 × platelets (x10^9^/L) – 0.66 × albumin (g/dL) (26). The FIB-4 was calculated using the following formula: age (years) × aspartate aminotransferase(U/L)/[platelets (x10^9^/L) × square root (alanine aminotransferase(U/L))] (27).

### Statistical Methods

Student's *t*-test was used for the comparison of normally distributed variables between the groups. For non-normally distributed data, the Mann-Whitney test was used. The relationship between sCD163/sMR and TE was analyzed by linear regression. Spearman's rank test was used to study the relationships of TE, sCD163, and sMR with histological scores. For differences in proportions, we used the χ^2^-test or Fisher's exact test.

Multiple ordered logistic regression was used to assess the relationship between the histological fibrosis score and TE, sCD163, and sMR. This analysis provides odds ratios (OR) describing the increase in the odds for a given fibrosis stage in a patient who has a specific increase in a parameter compared with another patient. We chose to present the results corresponding to a 25% increase in TE, sCD163 and sMR based on our previous experience (13, 28) and the distribution of these parameters according to fibrosis stages. We used multiple logistic regression analysis with given fibrosis stages (separate analysis for F ≥ 2, F ≥ 3, F4 stages) as the dependent variable and TE and sCD163/sMR as the explanatory to identify the best fitting models for predicting a given fibrosis stage, separately for the Italian and the Swedish cohort. The coefficients from this analysis were used as relative weights to compute the respective combined models. We then used the non-parametric Receiver Operating Characteristics (ROC) analysis to assess the performance of TE, sCD163/sMR, and their combinations in the prediction of fibrosis stages, followed by tests of equality of areas under the ROC curve (AUROCs) to compare the performance of individual markers and composite models. All data are expressed as means ± *SD* and medians with interquartile ranges (IQR) or proportions. A *p* ≤ 0.05 was considered statistically significant. STATA version 14.0^®^StataCorp LP was used for data analysis.

## Results

### Patients Characteristics

Demographic, clinical, biochemical, and histological data for the patients from both cohorts are shown in Table 1. The patients in the Swedish cohort were older, and a higher proportion had diabetes. Likewise, the Swedish patients had histologically more severe MAFLD, with more frequent steatohepatitis and advanced fibrosis stages. In addition, the levels of sCD163 and AST were also higher in the patients from the Swedish cohort.

**Table 1:** Patient characteristics.

	**Italian cohort**	**Swedish cohort**	***P***
	**(*****n*** **=** **141)**	**(*****n*** **=** **70)**	
Age (years)	43 ± 11	51 ± 14	<0.001
Sex [m/f (%)]	106 (75%)/35 (25%)	48 (69%)/22 (31%)	0.31
BMI (kg/m^2^)	28 ± 4	31 ± 4	<0.001
Diabetes [*n* (%)]	34 (24%)	19 (35%)	0.02
Steatohepatitis (FLIP algorithm) [*n* (%)]	78 (55%)	49 (70%)	0.04
**Fibrosis stage [*****n*** **(%)]**
0	50 (35%)	9 (13%)	<0.001
1	29 (21%)	26 (37%)	
2	30 (21%)	14 (20%)	
3	25 (18%)	8 (11%)	
4	7 (5%)	13 (19%)	
F ≥2, NAS ≥4 and steatohepatitis [*n* (%)]	31 (22%)	25 (36%)	0.03
sCD163 (mg/L)	1.6 (1.2–2.3)	3.4 (2.3–4.8)	<0.001
sMR (mg/L)	-	0.28 (0.22–0.39)	-
ALT (IU/L)	66 (42–94)	68 (47–114)	0.23
AST (IU/L)	36 (28–48)	47 (34–68)	<0.001
Albumin (g/L)	46 ± 4	39 ± 3	<0.001
Platelets (x10^9^/L)	230 ± 70	216 ± 57	0.16

*Parameters are presented as means ± SD for normally distributed and medians (interquartile range) for non-normally distributed variables, and as total number (%) for categorical variables*.

*BMI, Body Mass Index; FLIP, Fatty Liver Inhibition of Progression; sCD163, soluble CD163; sMR, soluble mannose receptor; ALT, alanine transaminase; AST, aspartate transaminase*.

### Associations of Macrophage Markers and TE With Fibrosis

**Figure 1:**
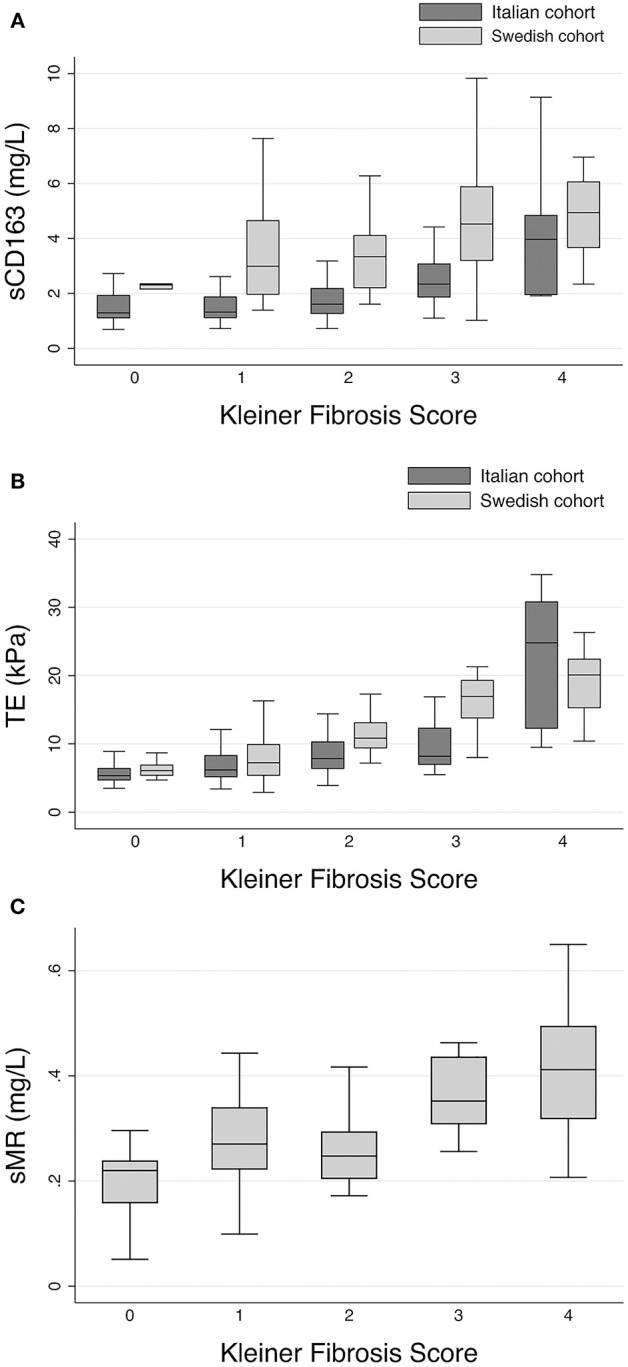
Associations of macrophage markers and transient elastography with fibrosis in the two cohorts. **(A)** sCD163 in the Italian and Swedish cohorts. Italian cohort, rho = 0.42, *p* < 0.001; Swedish cohort, rho = 0.43, *p* < 0.001. **(B)** TE in the Italian cohort and Swedish cohorts. Italian cohort, rho = 0.56, *p* < 001; Swedish cohort, rho = 0.73, *p* < 0.001. **(C)** sMR in the Swedish cohort, rho = 0.46, *p* < 0.001. Boxes represent interquartile ranges (IQR) with medians; whiskers show adjacent values. sCD163, soluble CD163; TE, transient elastography; sMR, soluble mannose receptor.

In the Italian cohort, sCD163 showed a moderate association with fibrosis (rho = 0.42, *p* < 0.001), as shown in Figure 1A. TE also correlated well with fibrosis (rho = 0.56, *p* < 0.001; Figure 1B). TE showed an even closer association with fibrosis in the Swedish cohort (rho = 0.73, *p* < 0.001; Figure 1B), which was better than for sCD163 (rho = 0.43, *p* < 0.001; Figure 1A), and sMR (rho = 0.46, *p* < 0.001; Figure 1C).

In the multiple ordered logistic regression analysis, both TE and sCD163 were significantly associated with the stage of fibrosis (OR = 1.05, *p* < 0.001 and OR = 1.13, *p* = 0.013, respectively) in the Italian cohort. In the same analysis including TE and sCD163 in patients from Sweden, TE showed a significant association (OR = 1.03, *p* = 0.002) and sCD163 showed a trend (OR = 1.05, *p* = 0.088). Similarly, the multivariate analysis with TE and sMR as the explanatory variables resulted in a significant association by TE (OR = 1.03, *p* = 0.004) and a trend by sMR (OR = 2.38, *p* = 0.062). When including both TE, sCD163, and sMR in the analysis in the Swedish cohort, TE remained significantly associated with fibrosis (OR = 1.03, *p* = 0.006), whereas sCD163 and sMR lost significance (OR = 1.03, *p* = 0.32 and OR = 1.91, *p* = 0.21, respectively).

### Prediction of Fibrosis by TE, Macrophage Markers, and Combined Models

In the ROC analysis, both TE and sCD163 showed good prediction of fibrosis in the Italian cohort, especially for F ≥ 3 and F4 stages. Combining TE and sCD163 resulted in slightly higher AUROCs than for TE alone, however, this improvement was not statistically significant (Table 2).

**Table 2:** Areas under the Receiver Operating Characteristics curve with 95% Confidence Intervals for fibrosis stages prediction by sCD163, sMR and transient elastography in the Italian and Swedish cohorts.

	**F ≥ 2**	**F ≥ 3**	**F4**
**Italian cohort**
sCD163	0.71 (0.63–0.80)	0.82 (0.74–0.90)	0.89 (0.76–1.0)
TE	0.79 (0.72–0.87)	0.81 (0.73 – 0.89)	0.95 (0.90–1.0)
TE + sCD163	0.79 (0.72–0.86)	0.85 (0.78–0.92)	0.97 (0.93–1.0)
**Swedish cohort**
sCD163	0.70 (0.56–0.82)	0.75 (0.62–0.88)	0.75 (0.62–0.89)
sMR	0.68 (0.56–0.81)	0.82 (0.71–0.92)	0.79 (0.66–0.93)
TE	0.88 (0.80–0.97)	0.90 (0.83–0.97)	0.87 (0.78–0.96)
TE + sCD163	0.87 (0.78–0.96)	0.90 (0.83–0.96)	0.86 (0.76–0.95)
TE + sMR	0.88 (0.79–0.96)	0.89 (0.81–0.97)	0.85 (0.74–0.95)
TE + sCD163 + sMR	0.87 (0.78–0.96)	0.89 (0.82–0.97)	0.84 (0.74–0.95)

*sCD163, soluble CD163; TE, transient elastography; sMR, soluble mannose receptor*.

**Figure 2:**
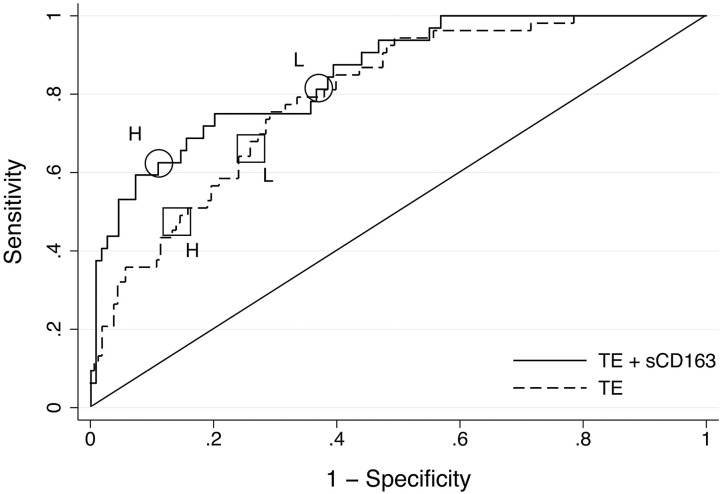
Receiver Operating Characteristics curves for transient elastography and combined transient elastography + sCD163 for advanced fibrosis (*F* ≥ 3) in the Italian cohort. Area under the ROC curve for transient elastography (TE) 0.81 (95% CI: 0.73–0.89), for the combined model 0.85 (95% CI: 0.78–0.92). The squares show the high (H) (9.6 kPa) and low (L) (7.9 kPa) cut-off values for TE, and the circles the high (3.72) and low (2.93) cut-off values for the combined model.

Nevertheless, we explored whether the numerically higher AUROC of the combined model translated into an improvement in the negative and positive predictive values (NPV and PPV). We thus determined the predictive characteristics of TE and the combined model (TE + sCD163) for advanced fibrosis (F ≥ 3) based on its importance for long-term outcome in MAFLD (4). We used the established cut-off values for TE (< 7.9 and ≥9.6 kPa) (29), and determined 2 cut-offs values of the combined model, a low cut-off for ruling out and a high cut-off for ruling in advanced fibrosis, based on the ROC curve (Figure 2). The combined model showed slightly higher NPV (92 vs. 88%) and PPV (62 vs. 50%) (Table 3).

**Table 3:** Predictive value of transient elastography and combined transient elastography + sCD163 for advanced fibrosis (F ≥ 3) in the Italian cohort.

**TE**	**Low cut-off**	**Indeterminable**	**High cut-off**	**Total**
	**(<7.9 kPa)**	**(7.9–9.6 kPa)**	**(≥9.6 kPa)**	
Total	92	19	30	141
F 0–2	81	13	15	109
F 3–4	11	6	15	32
Sensitivity	66%		47%	
Specificity	74%		86%	
Positive predictive value	43%		50%	
Negative predictive value	88%		85%	
**TE** **+** **sCD163**	**Low cut-off**	**Indeterminable**	**High cut-off**	**Total**
	**(≤2.93)**	**(2.93–3.46)**	**(≥3.72)**	
Total	75	34	32	141
F 0–2	69	28	12	109
F 3–4	6	6	20	32
Sensitivity	82%		62%	
Specificity	63%		89%	
Positive predictive value	41%		62%	
Negative predictive value	92%		89%	

*sCD163, soluble CD163; TE, transient elastography*.

In the Swedish cohort, the AUROCs of sCD163 and sMR were moderately good, whereas TE performed even better. Adding sCD163, sMR or both of these markers to TE failed to change its AUROCs (Table 2), for which reason we did not pursue the predictive values of the combined models.

### Prediction of Combined Significant Activity and Fibrosis

Besides fibrosis, the presence of necroinflammatory activity and steatohepatitis may be determinants of progressive disease and pharmacological response, and a combination of at least significant (≥F2) fibrosis, steatohepatitis and NAFLD activity score (NAS) ≥4 has been proposed to identify patients at risk and inclusion into clinical trials (9). In the Italian cohort, 22 percent of the patients had a combination of these features, and 36 percent in the Swedish cohort (Table 1). We tested the ability of macrophage markers and TE to predict this composite endpoint. Neither sCD163 nor sMR performed well, with AUROCs below 0.70. TE showed better prediction, but still with AUROCs lower than 0.80 in both cohorts (Table 4). Combining the parameters did not improve the AUROCs (Table 4). AST, which was included in the composite score in the report mentioned above (9), showed AUROCs similar to those of sCD163 (highest AUROC 0.69), and the addition of AST to TE did not improve its performance (highest AUROC 0.77).

**Table 4:** Areas under the Receiver Operating Characteristics curve with 95% Confidence Intervals for the prediction of combined fibrosis ≥2, NAFLD activity score ≥4 and steatohepatitis by sCD163, sMR, AST and transient elastography in the Italian and Swedish cohorts.

**Italian cohort**
sCD163	0.68 (0.57–0.78)
TE	0.78 (0.68–0.87)
TE + sCD163	0.77 (0.68–0.86)
**Swedish cohort**
sCD163	0.65 (0.51–0.79)
sMR	0.61 (0.47–0.75)
TE	0.76 (0.65–0.87)
TE + sCD163	0.75 (0.64–0.87)
TE + sMR	0.74 (0.62–0.85)
TE + sCD163 + sMR	0.76 (0.65–0.87)

*sCD163, soluble CD163; TE, transient elastography; sMR, soluble mannose receptor*.

Of note, sCD163 was not significantly higher in the patients with NASH [1.69 (1.19–2.4) vs. 1.52 (1.15–2.24) mg/L, *p* = 0.43] in the Italian cohort. In the Swedish cohort, this was the case for both sCD163 [3.59 (2.35–4.94) vs. 2.97 (1.97–4.61) mg/L, *p* = 0.31] and sMR [0.29 (0.24–0.39) vs. 0.25 (0.22–0.32) mg/L, *p* = 0.42]. Furthermore, looking at the histological grades of steatosis, lobular inflammation and hepatocyte ballooning, sCD163 was only significantly associated with steatosis in the Italian cohort, whereas sMR tended to associate with ballooning in the Swedish cohort. TE showed no significant correlation with any histological measures other than fibrosis in any cohort (Supplementary Tables 1, 2).

### Macrophage Markers in Relation to NAFLD Fibrosis Score and FIB-4

We calculated the established markers of liver fibrosis NAFLD Fibrosis Score and FIB-4 in both cohorts. In the Italian cohort, FIB-4 and NFS had higher AUROCs for F ≥ 2 compared with sCD163, whereas sCD163 had higher AUROCs for F ≥ 3 and F4 stages (Table 5). A combination of sCD163 and NFS resulted in an AUROC higher than both of these markers alone for F ≥ 2 fibrosis, and combinations of sCD163 with FIB-4 and NFS showed slightly higher AUROCS for F ≥ 3. The other possible combinations did not result in increasing AUROCs (Table 5).

**Table 5:** Areas under the Receiver Operating Characteristics curve with 95% Confidence Intervals for fibrosis stages prediction by sCD163, sMR, NAFLD Fibrosis Score and FIB-4 the Italian and Swedish cohorts.

	**F ≥ 2**	**F ≥ 3**	**F4**
**Italian cohort**
sCD163	0.71 (0.63–0.80)	0.82 (0.74–0.90)^*^	0.89 (0.76–1.0)
NFS	0.76 (0.68–0.85)	0.70 (0.59–0.81)	0.74 (0.40–1.0)
FIB-4	0.73 (0.65–0.82)	0.68 (0.57–0.79)	0.74 (0.44–1.0)
sCD163 + NFS	**0.80 (0.72–0.88)^‡^**	**0.83 (0.74–0.91)^†^**	0.81 (0.57–1.00)
sCD163 + FIB-4	**0.76 (0.68–0.84)**	0.83 (0.74–0.91)^*^	0.86 (0.69–1.00)
**Swedish cohort**
sCD163	0.70 (0.56–0.82)	0.75 (0.62–0.88)	0.75 (0.62–0.89)
sMR	0.68 (0.56–0.81)	0.82 (0.71–0.92)	0.79 (0.66–0.93)
NFS	0.64 (0.49–0.79)	0.75 (0.61–0.89)	0.87 (0.76–0.94)
FIB-4	0.74 (0.61–0.86)	0.85 (0.76–0.95)	0.92 (0.85–0.99)^‡^
sCD163 + NFS	0.66 (0.52–0.81)	**0.81 (0.68–0.94)**	0.88 (0.78–0.98)
sCD163 + FIB-4	**0.76 (0.64–0.87)**	0.86 (0.76–0.96)^‡^	0.91 (0.83–0.98)^‡^
sMR + NFS	0.63 (0.48–0.78)	0.79 (0.67–0.91)	0.87 (0.77–0.97)
sMR+ FIB-4	**0.76 (0.64–0.87)**	**0.89 (0.82–0.97)**	0.92 (0.85–0.99)^§^

*Combinations with possible synergy between markers in bold*.

*sCD163, soluble CD163; NFS, NAFLD Fibrosis Score; sMR, soluble mannose receptor*.

* *p < 0.05 compared with FIB-4*.

† *p < 0.05 compared with NFS*.

‡ *p < 0.005 compared with sCD163*.

§ *p < 0.05 compared with sMR*.

In the Swedish cohort, FIB-4 had higher AUROCs than both sCD163 and sMR for all fibrosis stages. Combining FIB-4 with sMR provided higher AUROCs for F ≥ 2 and F ≥ 3 stages than both these markers, whereas combinations with sCD163 did not result in additional predictive value.

NFS showed moderate prediction for fibrosis in the Swedish cohort, however, a combination of sCD163 and NFS had an AUROC for F ≥ 2 higher than both these markers alone (Table 5).

## Discussion

In this study of two independent cohorts of patients with biopsy-proven MAFLD and available TE, we measured macrophage markers sCD163 and sMR and combined them with TE hypothesizing that this would improve the prediction of fibrosis. The markers independently predicted fibrosis moderately well, but in contrast to our hypothesis, adding sCD163 and sMR to TE did not significantly improve its predictive capability.

Our study has strengths and limitations. The key strength was the well-characterized patients with histologically classified disease in two independent cohorts. Robust well-established and validated methods including the XL probe were used for the measurement of liver stiffness and for macrophage markers. The most significant limitation was the lower number of patients in the Swedish cohort, which inherently could raise the issue of inadequate power, however, as the combinations of TE with macrophage markers had generally lower AUROCs than TE alone, we do not believe that a larger number of patients in this cohort would have resulted in a better performance of the combined models. A more general drawback may be the cross-sectional design of the study and its focus on liver histology. Liver biopsy is an imperfect gold standard due to potential sampling error and interobserver variability, and the possible resulting bias is not easily predictable.

Combinations of elastography with biochemical markers and composite scores have been explored in MAFLD before. Gaia et al. showed no improvement in the fibrosis prediction of TE with the addition of ultrasonographic and biochemical measures in a smaller study (30). A larger study of MAFLD patients showed similar AUROCs for the FibroMeter™-TE combination and TE alone, however, with a markedly better PPV (31), followed by a more recent study demonstrating higher AUROCs for all fibrosis stages for the FibroMeter™-TE model (32). Similarly, another commercially available score, the Enhanced Liver Fibrosis (ELF™)-test, showed improved prediction when combined with TE (33). The readily available and highly validated models NAFLD fibrosis score (NFS) and FIB-4 have been investigated in sequential algorithms with TE resulting in the increase of correctly classified MAFLD patients (34–36).

In our study, sCD163 and sMR predicted fibrosis with moderate accuracy. Furthermore, both sCD163 and sMR showed independent associations with fibrosis in the multiple ordered logistic regression analysis with TE as a covariate. These findings supported our hypothesis and held promise of an add-on value of the macrophage markers for fibrosis prediction when combined with TE. Of note, in the Italian cohort the combination of sCD163 and TE did in fact result in numerically higher AUROCs than TE alone, as well as higher NPV and PPV for advanced fibrosis. However, the increase in the AUROCs was not significant and while the NPV of the combined model was strong at 92%, TE in itself already had an adequate NPV at 88%. At the same time, the PPV of the combined model remained low at 62%, a value not fit to rule in advanced fibrosis. In the Swedish cohort, the AUROCs of the combined models were even slightly lower compared to TE. Thus, based on our data, sCD163 and sMR did not contribute to the detection of advanced fibrosis beyond TE.

Several explanations for this result can be considered. In line with earlier studies, combining sCD163 and TE led to a higher PPV in the Italian cohort, which however was too low for practical use. This may partly be explained by the distribution of fibrosis in the Italian cohort with only 23% having fibrosis stage 3 or higher. The prevalence of disease affects predictive values, and in this case, favors NPV over PPV. Thus, sCD163 may have had a more pronounced impact on PPV in a cohort with more frequent advanced fibrosis. However, the distribution of fibrosis stages does not explain the similar AUROCs of TE and combined models in the patients from the Swedish cohort, as they had more severe fibrosis. The lack of an improvement of TE prediction by addition of sCD163 and sMR may be rooted in the nature of these biomarkers. sCD163 and sMR were not developed with the purpose of fibrosis detection, but are functional markers of macrophage activation, and have been associated with liver fibrosis reflecting the roles of macrophages in liver disease and fibrogenesis (10). In contrast, the method of TE to determine liver stiffness was identified and refined as a dedicated test of liver fibrosis, as were composite scores such as the NFS (26), the FibroMeter™ (37), and the ELF™ (38), which may explain that these markers may have a higher usability than the macrophage markers in terms of predictive values even with similar AUROCs. In this regard, it may prove fruitful to examine combinations of TE with other specific fibrosis markers, for instance the markers of collagen turnover that also have shown promising results in MAFLD (39).

Both sCD163 and sMR failed to help detect the combination of significant fibrosis, steatohepatitis and necroinflammation suggested as the FAST algorithm to identify patients with progressive disease suitable for clinical trials (9), and the levels of the macrophage markers were not significantly elevated in patients with steatohepatitis. In some of the other cohorts of MAFLD patients investigated by our group and others, sCD163 was higher in subjects with steatohepatitis, however, the association with fibrosis was considerably stronger (11–13). We have previously explained this finding by the higher variability in the histological assessment of features such as steatosis and inflammation compared to fibrosis (24), as well as by the dynamic nature of necroinflammatory activity, whereas fibrosis remains more stable over time. It is therefore not to be expected that macrophage markers can contribute to the detection of inflammatory activity in MAFLD. However, it is important to mention that the FAST algorithm will miss patients with advanced fibrosis but no steatohepatitis, whose prognosis is very similar to those who have both advanced fibrosis and steatohepatitis (4, 40), for which reason the detection of fibrosis is more appropriate for clinical practice.

In our study, the AUROCs of sCD163 were higher compared with NFS and FIB-4 in the Italian cohort, whereas FIB-4 was superior to sCD163 and sMR in the Swedish cohort. Interestingly, in several instances the combinations of the macrophage markers with NFS/FIB-4 showed AUROC values higher than the single markers, which suggests that sCD163 and sMR may be valuable as add-ons to the biomarker-based screening of MAFLD patients in the setting of primary care. However, this should be further tested in primary care cohorts more suitable for this purpose due to the expectedly lower prevalence of advanced fibrosis compared with our cohorts of patients referred to tertiary centers.

In conclusion, despite significant independent associations with fibrosis in two cohorts of patient with MAFLD, macrophage markers sCD163 and sMR did not improve the prediction of fibrosis by TE in combined models. This may be attributed to the nature of these markers as related to macrophage activation and not fibrosis *per se*, and other more fibrosis specific markers may be more useful and could be explored in future studies.

## Data Availability Statement

The raw data supporting the conclusions of this article will be made available by the authors, without undue reservation.

## Ethics Statement

The studies involving human participants were reviewed and approved by Ethics Committee of the University Hospital San Giovanni Battista of Torino and Regional Ethics Committee of Stockholm. The patients/participants provided their written informed consent to participate in this study.

## Author Contributions

KK: study design, conception, data analysis, interpretation, and manuscript preparation. CR, RY, AA, and HH: study subject inclusion, data acquisition, interpretation, and critical revision of the manuscript for intellectual content. HM: data analysis, interpretation, and critical revision of the manuscript for intellectual content. PS and EB: study design, conception, administrative support, data interpretation, and critical revision of the manuscript for intellectual content. HG: study design, conception, obtained funding, data interpretation, study supervision, and critical revision of the manuscript for intellectual content. All authors have approved the submitted manuscript.

## Conflict of Interest

RY was employed by the company Boehringer Ingelheim International, Gesellschaft mit beschränkter Haftung, Ingelheim, Germany. The remaining authors declare that the research was conducted in the absence of any commercial or financial relationships that could be construed as a potential conflict of interest.

## References

[1] Eslam M Sanyal AJ George J International Consensus Panel. MAFLD: a consensus-driven proposed nomenclature for metabolic associated fatty liver disease. Gastroenterology. (2020) 158:1999–2014.e1. 10.1053/j.gastro.2019.11.312 32044314

[2] Younossi Z Anstee QM Marietti M Hardy T Henry L Eslam M. Global burden of NAFLD and NASH: trends, predictions, risk factors and prevention. Nat Rev Gastroenterol Hepatol. (2018) 15:11–20. 10.1038/nrgastro.2017.109 28930295

[3] Taylor RS Taylor RJ Bayliss S Hagstrom H Nasr P Schattenberg JM. Association between fibrosis stage and outcomes of patients with nonalcoholic fatty liver disease: a systematic review and meta-analysis. Gastroenterology. (2020) 158:1611–25.e12. 10.1053/j.gastro.2020.01.043 32027911

[4] Angulo P Kleiner DE Dam-Larsen S Adams LA Bjornsson ES Charatcharoenwitthaya P. Liver fibrosis, but no other histologic features, is associated with long-term outcomes of patients with nonalcoholic fatty liver disease. Gastroenterology. (2015) 149:389–97. 10.1053/j.gastro.2015.04.043 25935633 PMC4516664

[5] Castera L. Diagnosis of non-alcoholic fatty liver disease/non-alcoholic steatohepatitis: non-invasive tests are enough. Liver Int. (2018) 38(Suppl.1):67–70. 10.1111/liv.13658 29427494

[6] Eddowes PJ Sasso M Allison M Tsochatzis E Anstee QM Sheridan D. Accuracy of fibroscan controlled attenuation parameter and liver stiffness measurement in assessing steatosis and fibrosis in patients with nonalcoholic fatty liver disease. Gastroenterology. (2019) 156:1717–30. 10.1053/j.gastro.2019.01.042 30689971

[7] European Association for the Study of the Liver European Association for the Study of Diabetes European Association for the Study of Obesity EASL-EASD-EASO clinical practice guidelines for the management of non-alcoholic fatty liver disease. J Hepatol. (2016) 64:1388–402. 10.1016/j.jhep.2015.11.004 27062661

[8] Honda Y Yoneda M Imajo K Nakajima A. Elastography techniques for the assessment of liver fibrosis in non-alcoholic fatty liver disease. Int J Mol Sci. (2020) 21:4039. 10.3390/ijms21114039 32516937 PMC7313067

[9] Newsome PN Sasso M Deeks JJ Paredes A Boursier J Chan WK. FibroScan-AST (FAST) score for the non-invasive identification of patients with non-alcoholic steatohepatitis with significant activity and fibrosis: a prospective derivation and global validation study. Lancet Gastroenterol Hepatol. (2020) 5:362–73. 10.1016/S2468-1253(19)30383-8 32027858 PMC7066580

[10] Kazankov K Jorgensen SMD Thomsen KL Moller HJ Vilstrup H George J. The role of macrophages in nonalcoholic fatty liver disease and nonalcoholic steatohepatitis. Nat Rev Gastroenterol Hepatol. (2019) 16:145–59. 10.1038/s41575-018-0082-x 30482910

[11] Mueller JL Feeney ER Zheng H Misdraji J Kruger AJ Alatrakchi N. Circulating soluble CD163 is associated with steatohepatitis and advanced fibrosis in nonalcoholic fatty liver disease. ClinTranslGastroenterol. (2015) 6:e114. 10.1038/ctg.2015.36 26448455 PMC4816035

[12] Kazankov K Tordjman J Moller HJ Vilstrup H Poitou C Bedossa P. Macrophage activation marker soluble CD163 and non-alcoholic fatty liver disease in morbidly obese patients undergoing bariatric surgery. J Gastroenterol Hepatol. (2015) 30:1293–300. 10.1111/jgh.12943 25772748

[13] Kazankov K Barrera F Moller HJ Rosso C Bugianesi E David E. The macrophage activation marker sCD163 is associated with morphological disease stages in patients with non-alcoholic fatty liver disease. Liver Int. (2016) 36:1549–57. 10.1111/liv.13150 27102725

[14] Bossen L Rebora P Bernuzzi F Jepsen P Gerussi A Andreone P. Soluble CD163 and mannose receptor as markers of liver disease severity and prognosis in patients with primary biliary cholangitis. Liver Int. (2020) 40:1408–14. 10.1111/liv.14466 32279422

[15] Gronbaek H Rodgaard-Hansen S Aagaard NK Arroyo V Moestrup SK Garcia E Macrophage activation markers predict mortality in patients with liver cirrhosis without or with acute-on-chronic liver failure (ACLF). J Hepatol. (2016) 64:813–22. 10.1016/j.jhep.2015.11.021 26639396

[16] Sandahl TD Stoy SH Laursen TL Rodgaard-Hansen S Moller HJ Moller S. The soluble mannose receptor (sMR) is elevated in alcoholic liver disease and associated with disease severity, portal hypertension, and mortality in cirrhosis patients. PLoS ONE. (2017) 12:e0189345. 10.1371/journal.pone.0189345 29236785 PMC5728513

[17] Hagstrom H Nasr P Ekstedt M Kechagias S Onnerhag K Nilsson E. Low to moderate lifetime alcohol consumption is associated with less advanced stages of fibrosis in non-alcoholic fatty liver disease. Scand J Gastroenterol. (2017) 52:159–65. 10.1080/00365521.2016.1239759 27650916

[18] Saunders JB Aasland OG Babor TF Delafuente JR Grant M Development of the alcohol-use disorders identification test (Audit) - who collaborative project on early detection of persons with harmful alcohol-consumption. 2. Addiction. (1993) 88:791–804. 10.1111/j.1360-0443.1993.tb02093.x 8329970

[19] Skinner HA Sheu WJ. Reliability of alcohol-use indexes - the lifetime drinking history and the mast. J Stud Alcohol. (1982) 43:1157–70. 10.15288/jsa.1982.43.1157 7182675

[20] Moller HJ Hald K Moestrup SK. Characterization of an enzyme-linked immunosorbent assay for soluble CD163. ScandJClinLab Invest. (2002) 62:293–9. 10.1080/003655102760145852 12476928

[21] Rodgaard-Hansen S Rafique A Christensen PA Maniecki MB Sandahl TD Nexo E. A soluble form of the macrophage-related mannose receptor (MR/CD206) is present in human serum and elevated in critical illness. Clin Chem Lab Med. (2014) 52:453–61. 10.1515/cclm-2013-0451 24114918

[22] Andersen ES Rodgaard-Hansen S Moessner B Christensen PB Moller HJ Weis N. Macrophage-related serum biomarkers soluble CD163 (sCD163) and soluble mannose receptor (sMR) to differentiate mild liver fibrosis from cirrhosis in patients with chronic hepatitis C: a pilot study. Eur J Clin Microbiol Infect Dis. (2014) 33:117–22. 10.1007/s10096-013-1936-3 24424890

[23] Moller HJ Soluble CD163. ScandJClinLab Invest. (2012) 72:1–13. 10.3109/00365513.2011.626868 22060747

[24] Kleiner DE Brunt EM Van NM Behling C Contos MJ Cummings OW. Design and validation of a histological scoring system for nonalcoholic fatty liver disease. Hepatology. (2005) 41:1313–21. 10.1002/hep.20701 15915461

[25] Bedossa P. Utility and appropriateness of the fatty liver inhibition of progression (FLIP) algorithm and steatosis, activity, and fibrosis (SAF) score in the evaluation of biopsies of nonalcoholic fatty liver disease. Hepatology. (2014) 60:565–75. 10.1002/hep.27173 24753132

[26] Angulo P Hui JM Marchesini G Bugianesi E George J Farrell GC. The NAFLD fibrosis score: a noninvasive system that identifies liver fibrosis in patients with NAFLD. Hepatology. (2007) 45:846–54. 10.1002/hep.21496 17393509

[27] Sterling RK Lissen E Clumeck N Sola R Correa MC Montaner J. Development of a simple noninvasive index to predict significant fibrosis in patients with HIV/HCV coinfection. Hepatology. (2006) 43:1317–25. 10.1002/hep.21178 16729309

[28] Kazankov K Barrera F Moller HJ Bibby BM Vilstrup H George J. Soluble CD163, a macrophage activation marker, is independently associated with fibrosis in patients with chronic viral hepatitis B and C. Hepatology. (2014) 60:521–30. 10.1002/hep.27129 24623375

[29] Wong VW Vergniol J Wong GL Foucher J Chan HL Le Bail B. Diagnosis of fibrosis and cirrhosis using liver stiffness measurement in nonalcoholic fatty liver disease. Hepatology. (2010) 51:454–62. 10.1002/hep.23312 20101745

[30] Gaia S Campion D Evangelista A Spandre M Cosso L Brunello F. Non-invasive score system for fibrosis in chronic hepatitis: proposal for a model based on biochemical, FibroScan and ultrasound data. Liver Int. (2015) 35:2027–35. 10.1111/liv.12761 25495478

[31] Loong TC Wei JL Leung JC Wong GL Shu SS Chim AM. Application of the combined FibroMeter vibration-controlled transient elastography algorithm in Chinese patients with non-alcoholic fatty liver disease. J Gastroenterol Hepatol. (2017) 32:1363–9. 10.1111/jgh.13671 27936280

[32] Ducancelle A Leroy V Vergniol J Sturm N Le Bail B Zarski JP. A single test combining blood markers and elastography is more accurate than other fibrosis tests in the main causes of chronic liver diseases. J Clin Gastroenterol. (2017) 51:639–49. 10.1097/MCG.0000000000000788 28692443

[33] Inadomi C Takahashi H Ogawa Y Oeda S Imajo K Kubotsu Y. Accuracy of the enhanced liver fibrosis test, and combination of the enhanced liver fibrosis and non-invasive tests for the diagnosis of advanced liver fibrosis in patients with non-alcoholic fatty liver disease. Hepatol Res. (2020) 50:682–92. 10.1111/hepr.13495 32090397

[34] Chan WK Nik Mustapha NR Mahadeva S. A novel 2-step approach combining the NAFLD fibrosis score and liver stiffness measurement for predicting advanced fibrosis. Hepatol Int. (2015) 9:594–602. 10.1007/s12072-014-9596-7 25788185

[35] Chan WK Treeprasertsuk S Goh GB Fan JG Song MJ Charatcharoenwitthaya P. Optimizing use of nonalcoholic fatty liver disease fibrosis score, fibrosis-4 score, and liver stiffness measurement to identify patients with advanced fibrosis. Clin Gastroenterol Hepatol. (2019) 17:2570–80.e37. 10.1016/j.cgh.2019.03.006 30876959

[36] Petta S Wong VW Camma C Hiriart JB Wong GL Vergniol J. Serial combination of non-invasive tools improves the diagnostic accuracy of severe liver fibrosis in patients with NAFLD. Aliment Pharmacol Ther. (2017) 46:617–27. 10.1111/apt.14219 28752524

[37] Cales P Boursier J Chaigneau J Laine F Sandrini J Michalak S. Diagnosis of different liver fibrosis characteristics by blood tests in non-alcoholic fatty liver disease. Liver Int. (2010) 30:1346–54. 10.1111/j.1478-3231.2010.02314.x 20666992

[38] Rosenberg WMC Voelker M Thiel R Becka M Burt A Schuppan D. Serum markers detect the presence of liver fibrosis: a cohort study. Gastroenterology. (2004) 127:1704–13. 10.1053/j.gastro.2004.08.052 15578508

[39] Daniels SJ Leeming DJ Eslam M Hashem AM Nielsen MJ Krag A. ADAPT: an algorithm incorporating PRO-C3 accurately identifies patients with NAFLD and advanced fibrosis. Hepatology. (2019) 69:1075–86. 10.1002/hep.30163 30014517

[40] Hagstrom H Nasr P Ekstedt M Hammar U Stal P Hultcrantz R. Fibrosis stage but not NASH predicts mortality and time to development of severe liver disease in biopsy-proven NAFLD. J Hepatol. (2017) 67:1265–73. 10.1016/j.jhep.2017.07.027 28803953

