# Supplementary material for: Macrophage Markers Do Not Add to the Prediction of Liver Fibrosis by Transient Elastography in Patients With Metabolic Associated Fatty Liver Disease
Source: Front Med (Lausanne). 2020 Dec 18;7:616212. doi: 10.3389/fmed.2020.616212 (PMC7775526; doi:10.3389/fmed.2020.616212)
Supplement: Supplementary file 1 [file Data_Sheet_1.docx]

Supplementary Table 1. Distribution of histological grades of steatosis, lobular inflammation and hepatocyte ballooning in the Italian and Swedish cohorts.

|  | **Italian cohort**  **(n = 141)** | **Swedish cohort**  **(n = 70)** | **P** |
| --- | --- | --- | --- |
| Steatosis (n (%))  0  1  2  3 | 6 (4 %)  71 (50 %)  38 (27 %)  26 (19 %) | 0 (0 %)  13 (19 %)  41 (59 %)  16 (22 %) | <0.001 |
| Lobular inflammation (n (%))  0  1  2  3 | 40 (28 %)  57 (40 %)  44 (31 %)  0 (0 %) | 8 (11 %)  32 (46 %)  24 (34 %)  6 (9 %) | <0.001 |
| Hepatocyte ballooning (n (%))  0  1  2 | 29 (21 %)  108 (76 %)  4 (3 %) | 20 (29 %)  33 (47 %)  17 (24 %) | <0.001 |

Parameters are presented as total number (%) for categorical variables.

Supplementary Table 2. Associations of sCD163, sMR and Transient Elastography with histological grades of steatosis, lobular inflammation and hepatocyte ballooning in patients from the Italian and Swedish cohorts.

Associations were tested using the Spearman correlation.

|  | **sCD163** | **sMR** | **TE** |
| --- | --- | --- | --- |
| Italian cohort | | | |
| **Steatosis** | rho=0.38, p<0.001 | - | rho=0.17, p=0.049 |
| **Lobular inflammation** | rho=0.07, p=0.38 | - | rho=0.02, p=0.78 |
| **Hepatocyte ballooning** | rho=0.07, p=0.40 | - | rho=0.11, p=0.19 |
| Swedish cohort | | | |
| **Steatosis** | rho=0.05, p=0.71 | rho=0.05, p=0.70 | rho= -0.06, p=0.62 |
| **Lobular inflammation** | rho=0.07, p=0.55 | rho=0.10, p=0.39 | rho=0.12, p=0.32 |
| **Hepatocyte ballooning** | rho=0.14, p=0.24 | rho=0.23, p=0.06 | rho=0.13, p=0.27 |

sCD163, soluble CD163; sMR, soluble mannose receptor; TE, transient elastography
